# The Bacillus of Whooping-Cough

**Published:** 1883-04

**Authors:** 


THE BACILLUS OF WHOOPING COUGH.—Dr. C. Burger, of Bonn, in the first number of the *Berliner Klinische Wochenschrift* for this year, describes at length the special micro-organisms of pertussis, which he states can be found in any specimen of whooping-cough sputum. They appear, under the immersion lens VII, ocular O of Seibert-Krafft, as small elongated elliptical bodies of unequal length, the smallest being double as long as broad. Under a very strong power, transverse subdivision can be detected in the longest specimens. They may form chains or groups, but are generally isolated and scattered singly all over the field. They bear a certain resemblance to *Leptothrix buccalis*, the spores of which are often found in whooping-cough sputum; but the latter are larger and stouter, and near them the filiform mature leptothrix is always present. Occasionally, some of the specific bacilli are found to be inside the mucus-cells in the sputum The bacillus is easily prepared; they can be readily recognized if colored in the usual way by watery solutions of aniline. Fuschin and methyl-violet were employed by Dr. Burger. As in the case of *Bacillus tuberculosis*, this micro-organism is best studied when mounted in the dry way. Dr. Burger concludes that this bacillus is the actual producer of pertussis, because it is not found in any other kind of sputum, because it is so abundantly produced in whooping-cough that its influence cannot be doubted, because its abundance increases in direct proportion with the severity of the disease, and "because the course and symptoms of the whole disease are best explained by the development of this fungus."—*British Medical Journal.*

Dr. Clark Bell, in his inaugural at the January meeting of the Medico-Legal Society, stated, among others, the following, as worthy of being brought before the Legislature: the question of reform in the laws relating to the office of Coroner, and the adoption of a new system of investigation and proceeding in all cases of sudden death, when reasonable cause exists for the belief that the death was by violence, or has resulted from other than natural causes. This question has been before this body for some time, and the Society stands committed in favor of a substantial and radical change in the existing law, and the appointment of medical men skilled in the investigation of such cases to take charge of the medical questions involved, and of having the legal questions determined by a Court of competent power and jurisdiction. The Medical Society of the State of New York, on the invitation of your Committee, at the session of 1881, memorialized the Legislature in favor of reformatory legislation of this character. A competent committee of this Society has this subject now under consideration, and has made a formal report. I recommend that the subject be again brought to the attention of the Legislature, and that the State Medical Society be again asked to co-operate with us in asking legislation favoring the necessary change. I also recommend that the Society instruct the Committee on Coroners to frame and report a bill to be submitted to the present Legislature, in accordance with the previous action of the Society and its Executive Committee.

